# Differential influence of *Streptococcus mitis* on host response to metals in reconstructed human skin and oral mucosa

**DOI:** 10.1111/cod.13668

**Published:** 2020-08-31

**Authors:** Lin Shang, Dongmei Deng, Sanne Roffel, Susan Gibbs

**Affiliations:** ^1^ Department of Preventive Dentistry, Academic Centre for Dentistry Amsterdam (ACTA) University of Amsterdam and Vrije Universiteit Amsterdam Amsterdam The Netherlands; ^2^ Department of Oral Cell Biology, Academic Centre for Dentistry Amsterdam (ACTA) University of Amsterdam and Vrije Universiteit Amsterdam Amsterdam The Netherlands; ^3^ Department of Molecular Cell Biology and Immunology Amsterdam UMC, Vrije Universiteit Amsterdam Amsterdam The Netherlands

**Keywords:** allergy, commensal bacteria, host microbiome, in vitro, innate immune response, metals, oral mucosa, reconstructed human gingiva, reconstructed human skin, skin

## Abstract

**Background:**

Skin and oral mucosa are continuously exposed to potential metal sensitizers while hosting abundant microbes, which may influence the host response to sensitizers. This host response may also be influenced by the route of exposure that is skin or oral mucosa, due to their different immune properties.

**Objective:**

Determine how commensal *Streptococcus mitis* influences the host response to nickel sulfate (sensitizer) and titanium(IV) bis(ammonium lactato)dihydroxide (questionable sensitizer) in reconstructed human skin (RHS) and gingiva (RHG).

**Methods:**

RHS/RHG was exposed to nickel or titanium, in the presence or absence of *S. mitis* for 24 hours. Histology, cytokine secretion, and Toll‐like receptors (TLRs) expression were assessed.

**Results:**

*S. mitis* increased interleukin (IL)‐6, CXCL8, CCL2, CCL5, and CCL20 secretion in RHS but not in RHG; co‐application with nickel further increased cytokine secretion. In contrast, titanium suppressed *S. mitis*–induced cytokine secretion in RHS and had no influence on RHG. *S. mitis* and metals differentially regulated TLR1 and TLR4 in RHS, and predominantly TLR4 in RHG.

**Conclusion:**

Co‐exposure of *S. mitis* and nickel resulted in a more potent innate immune response in RHS than in RHG, whereas titanium remained inert. These results indicate the important influence of commensal microbes and the route of exposure on the host's response to metals.

## Graphical abstract

Host response to *S. mitis* and metals.

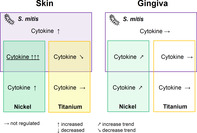

## 1 INTRODUCTION

The skin and oral mucosa form a defense barrier between the external environment and the human body. They are both capable of hosting abundant microbes, responding to environmental assault, and maintaining immune homeostasis.^1^ However, when exposed to sensitizers, they show very different properties, with the skin being immune stimulatory and the oral mucosa being tolerogenic. For example, first exposure to nickel—a common contact sensitizer—may cause sensitization in skin, which upon repeated exposure will result in allergic contact dermatitis (ACD).^2^ In contrast, if the first exposure to nickel is via the oral mucosa (eg, by dental retaining wires), tolerance may occur to further nickel exposure on the skin or mucosa, thus preventing sensitization.^3^, ^4^ The different responses of the skin and oral mucosa to sensitizers are reported to be influenced by various factors, including the tissue structure, innate immune properties, and the infiltration and migration of immune cells.^1^, ^5^ The central event in immune sensitization is the presentation of antigen by dendritic cells (DCs) to antigen‐responsive T cells in the local lymph node, which results in T‐cell priming (memory). The threshold for sensitization is now thought to be tightly regulated by the activation and maturation state of DCs and their cytokine and chemokine products, but also molecules secreted by local keratinocytes and fibroblasts.^6^, ^7^ Surprisingly little is known about the mechanisms by which local microbes influence sensitization, even though commensal microbes have been shown to play an important role in gut tolerance^8^, ^9^, ^10^ and oral tolerance,^11^, ^12^ and dysbiotic microbiota has been found to be related to skin allergy.^13^, ^14^

Nickel is classically regarded as a contact sensitizer because nickel allergy on the skin and oral mucosa is frequently reported.^15^ However, nickel is not always scored as a sensitizer in current assays and the reason is unknown. Nickel is identified as a moderate sensitizer in assays in vitro, including DCs, for example, h‐CLAT assay (human Cell Line Activation Test), LCSA assay (Loose‐Fit Coculture‐Based Sensitization Assay),^16^, ^17^ reconstructed human skin (RHS) with integrated Langerhans cells (LCs),^18^ and skin explant with T cells and monocyte‐derived dendritic cells.^19^ However in keratinocyte‐based assays without DCs, nickel scores as a nonsensitizer for example in KeratinoSens assay^20^ and reconstructed human epidermis (RHE) using interleukin (IL)‐18 as readout for sensitizer potency.^21^ Local lymph node assay (LLNA) in mice indicated nickel as a nonsensitizer, but this score was considered to be underpredictive^22^ due to the species‐specific mechanisms for nickel allergy between humans and mice.^23^ Furthermore, nickel allergy may also be tissue specific. Our previous studies using reconstructed human skin (RHS) and reconstructed human gingiva (RHG) with integrated LCs showed different host tissue responses to nickel. LC migration from the epithelium into the hydrogel was observed in both RHS and RHG.^24^ However, LC migration in RHS was CXCL12 dependent in line with the classical sensitization process, whereas LC migration in RHG was CXCL12 independent, illustrating the significant difference in innate immune mechanisms between the two tissues.^25^

Allergic manifestations to titanium‐based implants after orthopedic or dental surgery are also being increasingly reported.^26^ However, the current clinical tests used to diagnose titanium allergy are not always reliable: The result of the patch test can be influenced by the different solubility and penetration ability of the titanium salt used for the test, and the lymphocyte transformation test (LTT) and memory lymphocyte immunostimulation assay (MELISA) showed a low specificity regarding lymphocyte proliferation needing further optimization.^27^ Further contradicting reports from in vitro assays describe titanium as being inert; for example, titanium scored as a very weak irritant and nonsensitizer in the reconstructed human epidermis assay with IL‐18 release as readout,^21^ whereas titanium was described to have sensitization and irritation potentials by triggering host innate immune responses in pulmonary macrophages (mice and human) and keratinocytes (human)^28^, ^29^ as well as in intestine and liver (rat).^30^ Therefore, it is currently unclear whether the titanium‐related complaints observed in the clinic—which appear as edema, erosions, ulcers, or lichenoid lesions on the oral mucosa or skin—are due to allergy to titanium or a localized inflammation caused by mechanical load and cytotoxic leachables from titanium or titanium alloys.^7^, ^31^, ^32^

Because microbes trigger innate immune responses similar to those triggered by metals, it can be expected that microbes will also influence sensitization to metals. Furthermore, emerging evidence suggests that microbes maintain a symbiotic relationship with the host and influence both physiological and pathological host events via a group of host receptors known as the toll‐like receptors (TLRs). TLRs have been repeatedly shown to participate in a broad range of host events: nickel‐induced allergy,^2^, ^23^, ^33^, ^34^, ^35^ titanium‐related allergic responses,^36^ healthy‐associated host‐microbe interactions,^37^, ^38^, ^39^ and sensitization induced by the co‐exposure of metal and the microbial lipopolysaccharide (LPS).^40^, ^41^ Previously, we used RHG as a representative for healthy gingiva and showed that the oral commensal bacteria had a beneficial effect on host barrier function and increased the release of protective cytokines via the activation of the TLR signaling pathway.^38^, ^42^ Pathologically, a dysbiotic microbiome is recognized as a key determinant of immune dysregulation, and associated with a broad spectrum of intestinal allergic disorders.^8^, ^9^, ^10^ Other allergy‐related diseases such as asthma, eczema, and allergic rhinitis were found to coincide with the presence of a commensal/opportunistic pathogen, *Staphylococcus aureus*.^43^ In addition, LPSs were found to enhance the innate immune response of human monocyte‐derived dendritic cells to dental cast alloys.^44^ LPS‐activated TLR4 expression and the subsequent innate immune responses were further suggested necessary for inducing nickel allergy on the ear (skin) of mice, even in T cell–deficient mice.^40^, ^45^ Considering the different immune properties between the native skin and mucosa under the influence of microbes,^1^ it is highly possible that the local microbes contribute to the immune response during metal exposure and in doing so modulate the sensitization‐vs‐tolerance balance properties of these two tissues. However, none of the in vitro studies that evaluate potential sensitizers have yet incorporated the influence of living microbes on sensitization.

The aim of this study is to determine how commensal bacteria influence the response of skin and oral mucosa to potential metal sensitizers in vitro. We exposed RHS and RHG, consisting of a stratified differentiated epithelium on a fibroblast‐populated dermis/laminal propria (collagen hydrogel), to a mixture of metal and bacteria: nickel (II) sulfate hexahydrate or titanium(IV) bis(ammonium lactato)dihydroxide was co‐applied with *Streptococcus mitis*, a facultative commensal bacteria found on skin and on oral mucosa.^46^, ^47^ Thereafter, we investigated the host response by means of tissue morphology, viability, cytokine secretion, and TLR expression.

## 2 MATERIALS AND METHODS

### 2.1 Reconstructed human skin (RHS) and gingiva (RHG)

Human neonatal foreskin was obtained after informed consent from patients undergoing routine surgical procedures. Human non‐inflamed gingival tissue was obtained from healthy donors undergoing dental implant surgery or wisdom tooth extraction. Skin and gingiva were used anonymously and in accordance with the “Code for Proper Use of Human Tissue” as formulated by the Dutch Federation of Medical Scientific Organizations. Procedures are approved by the local medical research ethics committee of the Amsterdam UMC.

RHS and RHG were constructed exactly as described previously.^25^, ^48^ Keratinocytes (0.5 × 10^6^ cells) were seeded onto fibroblast‐populated collagen hydrogels in a 24 mm diameter transwell (pore size 0.4 μm, Corning, New York) and cultured submerged for 3 days. To induce epithelial differentiation, the cultures were then lifted to an air‐liquid interface and cultured for an additional 10 days. Twenty‐four hours before exposure and also at the time of exposure, the cultures were refreshed with medium without penicillin‐streptomycin or hydrocortisone. Cultures were incubated at 37°C, 7.5% CO_2_, and culture medium was refreshed twice a week.

### 2.2 *Staphylococcus mitis* growth condition

*S. mitis* LMG 14557 was cultured anaerobically at 37°C in a modified semi‐defined medium (pH 7.0) prepared exactly as described previously, in the presence of 1% glucose.^49^ Three days before exposure, *S. mitis* was cultured overnight for 24 hours, and then the pre‐culture was diluted into 1:1000 for another 16 hours growth until within the exponential phase (OD_600_ around 0.5). OD_600_ value was measured using SpectraMax Plus 384 (Molecular Devices, San Jose, California). The number of colony forming units (CFUs) of *S. mitis* at the time of exposure was determined by viable bacterial cell counting (CFU/mL): A sample was taken from the prepared *S. mitis* exposure, serial dilutions were made and plated on the brain hart infusion (BHI) agar plates, and colonies were counted after 96 hours of anaerobic incubation at 37°C.

### 2.3 Chemicals and *S. mitis* exposure

Titanium(IV) bis(ammonium lactato)dihydroxide solution (TiALH, CAS no. 65104‐06‐5, Sigma‐Aldrich, St. Louis, Missouri) and nickel (II) sulfate hexahydrate (NiSO4, CAS no. 10101‐97‐0) were used at the following concentrations: titanium: 20 mg/mL (68 mM) and 40 mg/mL (136 mM); nickel: 3 mg/mL (20 mM) and 15 mg/mL (97 mM). These concentrations were selected because they showed no more than a 5% decrease in metabolic activity (by MTT 3‐(4,5‐dimethylthiazol‐2‐yl)‐2,5‐diphenyltetrazolium bromide assay) in reconstructed human epidermis, compared to unexposed cultures.^21^

Exposure to chemicals and *S. mitis* was performed as follows: sterile gauze filters (03‐150/38, 12 mm diameter, Sefar Nitex, Heiden, Switzerland) were placed on top of the RHS or RHG cultures. Then 25 μL of prepared mixture of the following four combinations were applied onto the center of the filters: (a) Hank's Balanced Salt Solution (HBSS, Sigma‐Aldrich) as control; (b) 10^9^ CFU/mL of *S. mitis* in HBSS; (c) nickel or titanium at two different concentrations as indicated above; (d) 10^9^ CFU/mL of *S. mitis* mixed with nickel or titanium at two concentrations. The impregnated filters were kept on the cultures for 24 hours at 37°C, 7.5% CO_2_, and 95% humidity. To control the amount of *S. mitis* in the mixtures, CFU counting was performed as described above (section: *S. mitis* growth condition).

After 24 hours exposure, the viability of the applied *S. mitis* (by CFU counting) and RHS/RHG (by MTT assay) was determined. *S. mitis* samples were taken from the surface of the RHS or RHG using a sterile microbrush. Together with the filter, the brushed samples were sonicated and plated out on BHI agar plates for CFU counting as described above. The viability of RHS and RHG was measured by MTT assay as described previously.^50^ In short, a 3 mm diameter biopsy was taken from each culture and incubated with MTT (Sigma, 2 mg/mL dissolved in phosphate‐buffered saline [PBS]) overnight, and the absorbance was measured at 570 nm using a spectrophotometer (Mithras LB 940, Berthold Technologies, Bad Wildbad, Germany).

### 2.4 (Immuno)histochemistry and fluorescence in situ hybridization

RHS and RHG samples were fixed in 4% paraformaldehyde and embedded in paraffin. As previously described,^48^, ^51^ sections (5 μm) were cut and stained with hematoxylin and eosin (H&E) and Ki67. The Ki67 proliferation index (expressed as percentage) was determined by counting the number of Ki67 positive cells from 100 cells at four random locations in epithelial basal cell layer. Fluorescence in situ hybridization (FISH) was performed on paraffin sections (5 μm) according to the kit instructions (10MEH000, Ribo Technologies, Groningen, The Netherlands). The sections were further counterstained and sealed using fluoroshield mounting medium with DAPI (4',6‐diamidino‐2‐phenylindole, ab104139, Abcam, U.K.). Images were taken using a fluorescence microscope (Nikon Eclipse 80i microscope with Nikon Plan Fluor 20×/0.50 and 40×/0.75 objectives).

### 2.5 ELISA

Culture supernatants were collected at the time of harvesting and secretion of proteins determined by commercially available sandwich enzyme‐linked immunosorbent assays (ELISAs). For IL‐6, IL‐10, CCL2, CCL5, and CCL20, antibodies and recombinant proteins were purchased from R&D Systems (Minneapolis, Minnesota). For CXCL8, CXCL12, and IL‐18, ELISA kits were used (CXCL8: Sanquin, Amsterdam, The Netherlands; CXCL12: R&D Systems; IL‐18: MBL, Nagoya, Japan).

### 2.6 RNA and protein isolation

RHS or RHG epithelium were carefully removed from the fibroblast‐populated collagen hydrogel and washed with PBS. Next, total RNA and protein were isolated from the epithelium using a AllPrep RNA/Protein Kit (Qiagen, Hilden, Germany) following the manufacturer's instructions. Isolated protein was further precipitated using methanol and resuspended in complete lysis‐M buffer (Sigma‐Aldrich). Thereafter, the amount of protein was measured using the Pierce BCA Protein Assay Kit (Thermo Fisher Scientific, Waltham, Massachusetts), and RNA was measured using Nanodrop ND‐1000 Spectrophotometer (Thermo Fisher Scientific). The RNA and protein samples were stored at −80°C before further process.

### 2.7 Quantitative reverse transcriptase PCR

Genomic DNA elimination and complementary DNA (cDNA) synthesis were performed using RT^2^ First Strand Kit (Qiagen). Quantitative Reverse Transcriptase PCR (qRT‐PCR) reactions were performed on a ViiA 7 Real‐Time PCR System (Applied Biosystems, New York) using Fast SYBR Green Master Mix (Applied Biosystems) and paired primers (human TLRs 1‐6: HP206812, HP206813, HP206814, HP226301, HP206816, HP209082, Glyceraldehyde 3‐phosphate dehydrogenase (GAPDH): HP205798, OriGene Technologies, Rockville). Individual gene expression levels were normalized to GAPDH expression levels, and shown in fold‐change compared to the RHS or RHG control.

### 2.8 Western blotting

As described previously, the isolated proteins were prepared and separated on 4%‐12% Bis‐Tris Plus Gel (Invitrogen) and transferred to a Polyvinylidene fluoride (PVDF) membrane (iBlot 2 Transfer Stacks, Invitrogen). The membranes were blocked with 2% Bovine Serum Albumin (BSA) in Phosphate Buffered Saline with Tween 20 (PBST) for 1 hour and incubated with antibodies against TLR1, 2, 3, 5, and 6 (1:1000, Novus Biologicals, Littleton, Colorado); TLR4 (1:200, Santa Cruz Biotechnology, Dallas, Texas); and tubulin (1:1000, Abcam, Cambridge, United Kingdom) overnight at 4°C. Thereafter, membranes were washed three times in PBST and further incubated with infrared dye‐conjugated secondary antibodies against mouse (1:7500 for TLR3, 4, or 5) or against rabbit (1:7500 for TLR1, 2, 6, or tubulin). After washing, the blots were visualized using Sapphire Biomolecular Imager (Azure biosystems, Dublin, California) according to the manufacturer's instruction.

### 2.9 Statistics

Differences between *S. mitis* unexposed and exposed RHS and RHG were compared using unpaired *t‐*tests. For the dose‐dependent effect of nickel or titanium, differences were compared using one‐way analysis of variance (ANOVA) followed by Bonferroni's multiple‐comparisons test. Statistics were performed using GraphPad Prism version 7.0. Data represent the mean ± standard error of the mean (SEM) of three independent experiments, each performed in duplicate, and consisted of a different skin or mucosa donor (not patient patched) and a different *S. mitis* inoculum. Differences were considered significant when the *P*‐value was < .05; *denoting *P* < .05, **P < .01, and ***P < .001. Data are represented as mean ± SEM.

## 3 RESULTS

### 3.1 Co‐exposure of *S. mitis* with metal does not influence RHS or RHG viability

**FIGURE 1:**
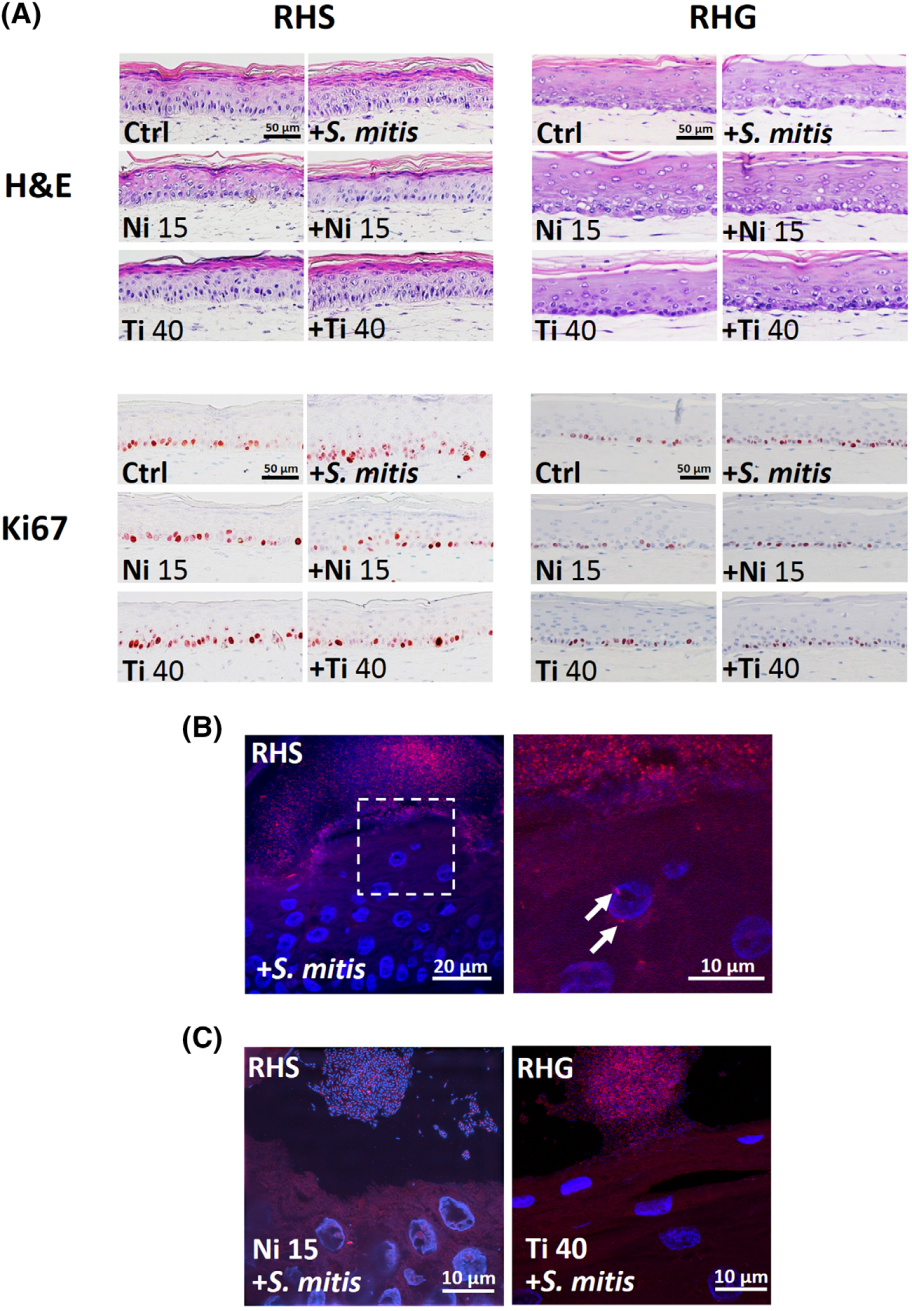
Co‐exposure of *Streptococcus mitis* and metals onto reconstructed human skin (RHS) and gingiva (RHG). (**A**), Histology (hematoxylin and eosin) and keratinocyte proliferation (Ki67‐positive nuclei, red) of RHS (left panel) and RHG (right panel) after exposure to *Streptococcus mitis* and metal. (**B**), Representative fluorescence in situ hybridization (FISH) staining on 5 μm paraffin sections shows presence of *S. mitis* bacterial ribosomal RNA (rRNA) on top of, and within the epithelium of, RHS (FISH in red and DAPI in blue, see arrows). (**C**), The presence of *S. mitis* when co‐applied with nickel (left panel) or titanium (right panel)

Exposure conditions have no detrimental effect on RHS or RHG histology. Both the RHS and RHG featured a stratified, differentiated epithelium on a fibroblast‐populated collagen hydrogel (Figure 1A). Similar as the in vivo tissues, RHS had a characteristic stratum corneum representative of the orthokeratinized skin, whereas RHG had a characteristic parakeratinized epithelium with nuclei being observed in the most differentiated outermost cell layers. Furthermore, RHG epithelium was thicker than RHS epithelium again in line with the native tissues.^52^ Ki67‐positive proliferating keratinocytes were observed in the basal layer of both RHS and RHG (Figure 1A).

**FIGURE 2:**
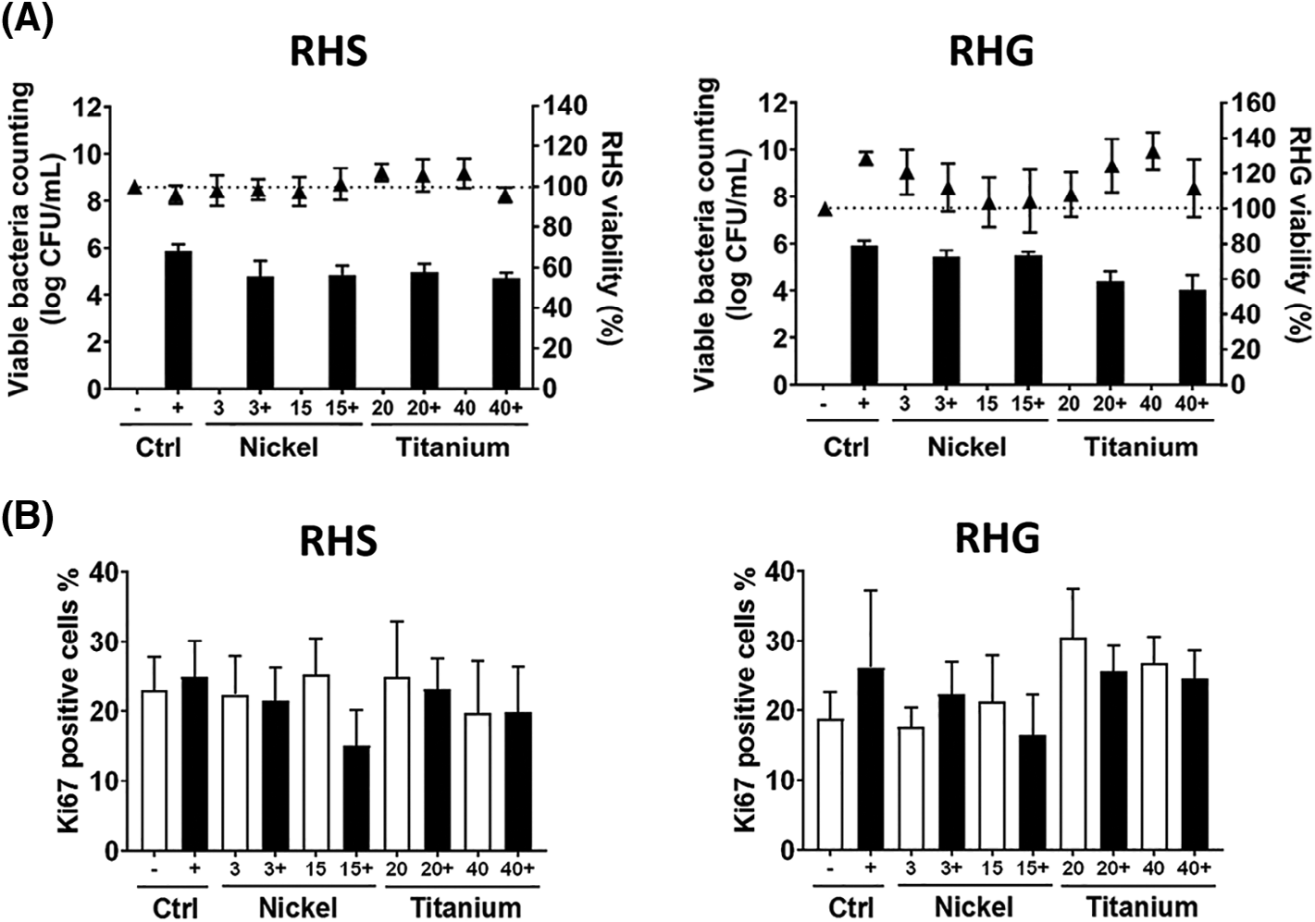
Viability of reconstructed human skin (RHS), gingiva (RHG), and *Streptococcus mitis* after 24 hours of exposure. (**A**), Viability of *Streptococcus mitis* and *S. mitis* co‐applied with metals are shown by the *S. mitis–*viable counts (left y‐axis; bars on graph). RHS or RHG viability compared to the vehicle (Hank's Balanced Salt Solution, HBSS) exposed culture is shown by the readings of MTT assay (right y‐axis; triangle symbols on graph). (**B**), The percentage of Ki67‐positive cells in the epithelial basal cell layer of RHS and RHG (metal alone: white bar, metal co‐application with *S. mitis*: black bar). All data represent the mean ± standard error of the mean (SEM) of three independent experiments, each performed in duplicate. Each experiment consisted of a different skin or mucosa donor (not patient patched) and a different *S. mitis* inoculum. *Statistics*: multiple *t*‐test with correction. Differences were considered significant when *P* < .05

Fluorescence in situ hybridization staining (FISH) showed the *S. mitis* rRNA (in red) present in the form of biofilms on top of both RHS and RHG and also sparingly within the epithelium (DAPI‐stained keratinocyte nuclei and *S. mitis* DNA in blue). As examples, RHS exposed to *S. mitis* (Figure 1B), RHS exposed to *S. mitis* and nickel, and RHG exposed to *S. mitis* and titanium are shown (Figure 1C). After 24 hours of exposure, no significant difference was found in the amounts of viable *S. mitis* retrieved from RHS or RHG in the presence or absence of metals (Figure 2A). No significant change in RHS or RHG viability or epithelial proliferation was observed when cultures were exposed to *S. mitis*, nickel, or titanium alone (Figure 2A), or when co‐exposed to *S. mitis* and nickel or titanium (Figure 2B).

### 3.2 *S. mitis* increases basal cytokine secretion in RHS but not RHG

**FIGURE 3:**
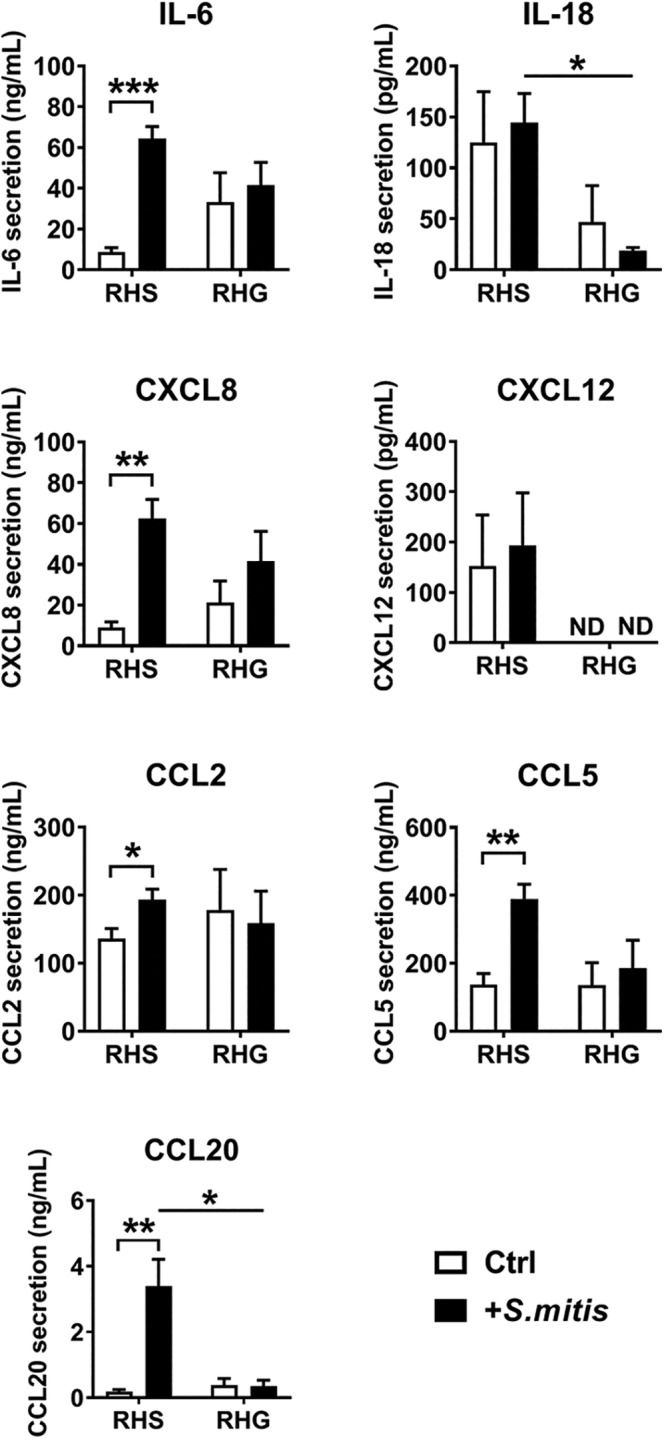
Influence of *Streptococcus mitis* on cytokine release in reconstructed human skin (RHS) and gingiva (RHG). After 24 hours of *Streptococcus mitis* exposure, cytokine and chemokine release from RHS and RHG were determined by enzyme‐linked immunosorbent assay (ELISA). IL‐10 release was below the detection limit of ELISA (12.5 pg/mL; data not shown). Data represent the mean ± standard error of the mean (SEM) of three independent experiments in duplicate. Each experiment used a different skin or mucosa donor and a different *S. mitis* inoculum. White bar, unexposed RHS/RHG; black bar, *S. mitis–*exposed RHS/RHG. ND, not detectable. *Statistics*: multiple *t*‐tests with correction. Differences were considered significant when *P* < .05. *P < .05; **P < .01; ***P < .001

To investigate the influence of *S. mitis* on RHS and RHG, pro‐inflammatory and antimicrobial cytokine secretion was determined (Figure 3). After *S. mitis* exposure, IL‐6, CXCL8, CCL2, CCL5, and CCL20 secretion increased from RHS but not from RHG. IL‐18 and CXCL12 were not regulated by *S. mitis* exposure in either RHS or RHG. In line with our previous findings, IL‐18, a keratinocyte‐derived chemokine that is related to contact sensitizer potency in reconstructed human skin epidermis,^21^ and CXCL12, a chemoattractant that is pivotal in mediating LCs migration during skin sensitization,^48^ were found to be higher in RHS than in RHG regardless of the presence of *S. mitis*.

### 3.3 Nickel‐mediated cytokine secretion is enhanced by *S. mitis* in RHS but not in RHG, while titanium suppresses cytokines induced by *S. mitis* in RHS

**FIGURE 4:**
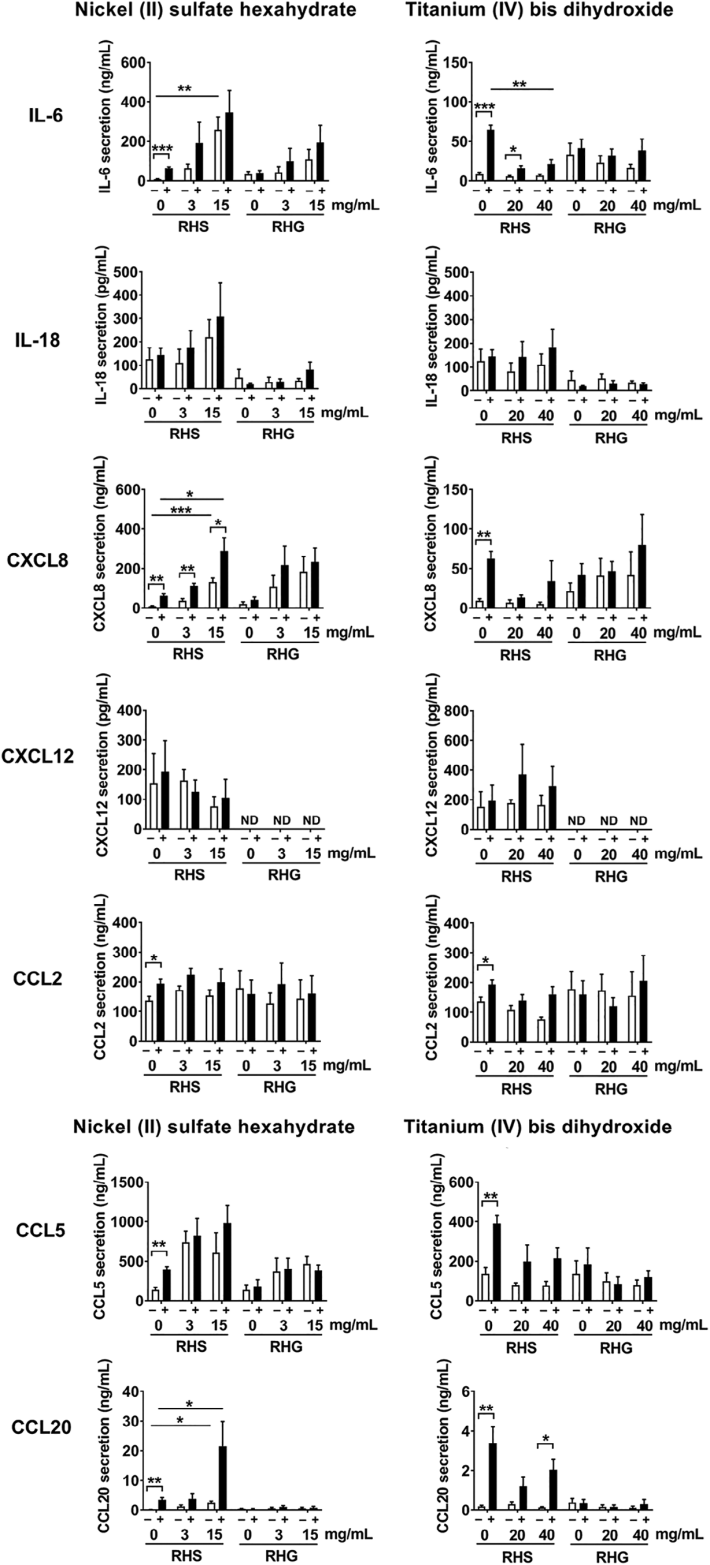
Influence of *Streptococcus mitis* and metal exposure on cytokine secretion. Reconstructed human skin (RHS) and gingiva (RHG) were exposed to nickel (II) sulfate hexahydrate (left panel, 3 and 15 mg/mL) or titanium(IV) bis(ammonium lactato)dihydroxide (right panel, 20 and 40 mg/mL) in the presence or absence of *S. mitis* for 24 hours (metal alone: white bar, metal co‐applied with *S. mitis*: black bar). ND, not detectable. The cytokine secretion in culture supernatants was determined by enzyme‐linked immunosorbent assay (ELISA). Data represent the mean ± standard error of the mean (SEM) of three independent experiments in duplicate, each experiment being with a different skin or mucosa donor (not patient patched) and each experiment with a different *S. mitis* inoculum. *S. mitis* exposed and unexposed results were compared by multiple *t*‐tests with correction. The dose‐dependent effect of nickel or titanium was determined using one‐way analysis of variance (ANOVA) followed by Bonferroni's multiple comparisons test. Differences were considered significant when *P* < .05. *P < .05; **P < .01; ***P < .001

Next it was determined whether co‐application of *S. mitis* together with nickel or titanium further influenced cytokine secretion (Figure 4). Exposure to nickel alone resulted in a dose‐dependent increase in IL‐6, CXCL8, CCL5, and CCL20 secretion from RHS (RHG: trend observed for IL‐6, CXCL8 and CCL5 secretion). Notably, *S. mitis* further increased this nickel‐dependent increase in cytokine secretion from RHS, particularly in the case of CXCL8 and CCL20 secretion. No such further increase was observed for RHG. IL‐18, CXCL12, and CCL2 secretion from RHS or RHG was not influenced by nickel in the presence or absence of *S. mitis*.

In contrast to nickel, exposure of titanium to RHS or RHG did not result in a dose‐dependent increase in cytokine secretion. Notably, titanium even suppressed the *S. mitis*–mediated cytokine secretion (IL‐6, CXCL8, CCL20) from RHS. Titanium, in the presence or absence of *S. mitis*, did not influence cytokine secretion from RHG. IL‐1α, IL‐1β, IL‐10, and CCL28 levels were below the detection limit of the ELISA in both RHS and RHG in all experimental conditions (data not shown). Taken together, these results indicate that nickel induces innate cytokine release, which is further enhanced by *S. mitis* in RHS but not in RHG. In contrast, titanium is inert and even suppresses *S. mitis*–mediated cytokine release from RHS.

### 3.4 Influence of *S. mitis* and metal co‐exposure on TLR expression

**FIGURE 5:**
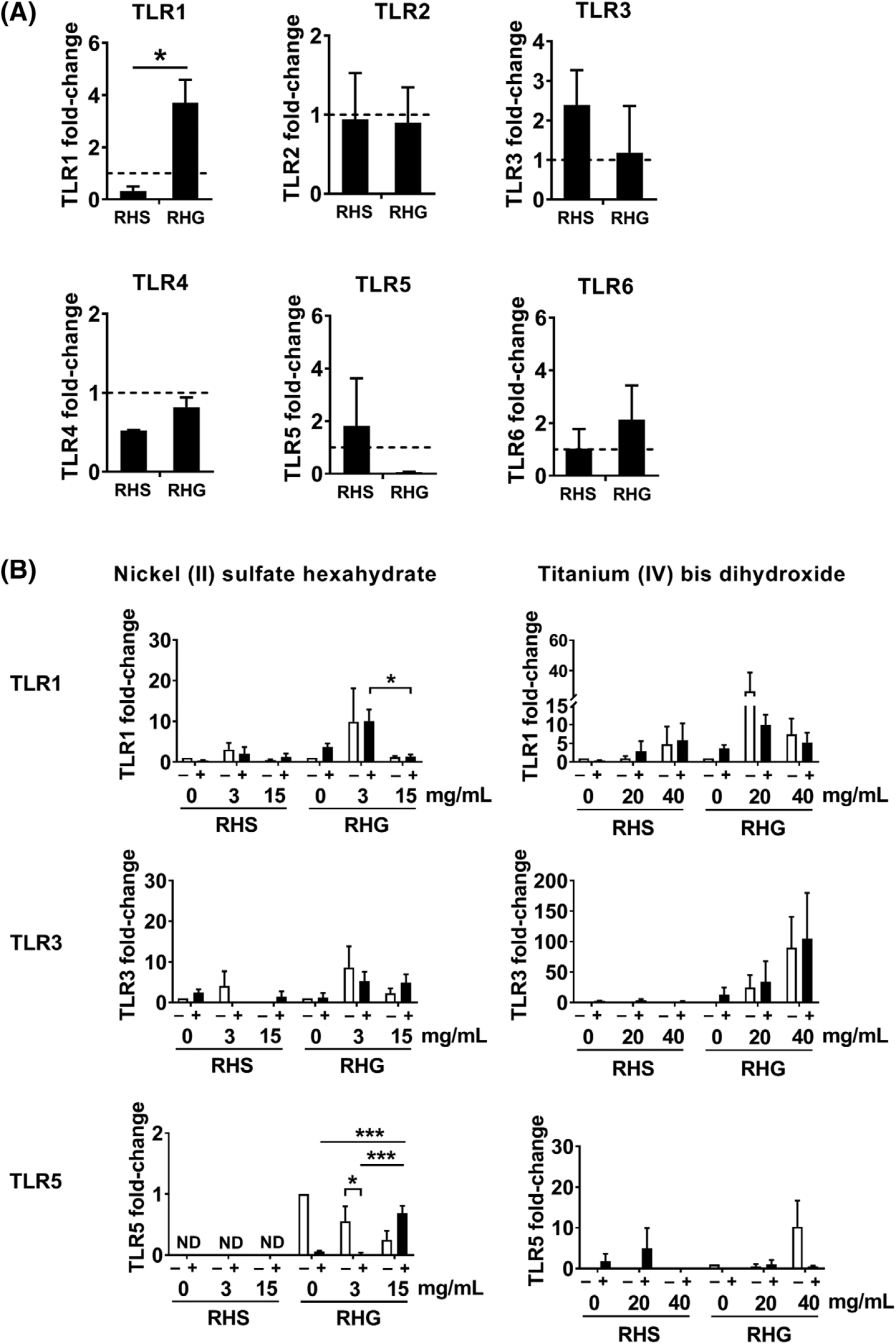
Toll‐like receptor (TLR) transcription in reconstructed human skin (RHS) and gingiva (RHG). (**A**), Relative TLR messenger RNA (mRNA) expression in RHS and RHG after *Streptococcus mitis* exposure and (**B**), after co‐exposure with nickel or titanium was determined by quantitative reverse transcriptase PCR (qRT‐PCR). The mRNA expression of TLR2, 4, and 6 was below the detectable level (data not shown). Data are all expressed as relative to the vehicle exposed RHS or RHG, and represent the mean ± standard error of the mean (SEM) of three individual experiments in duplicate. RHS and RHG exposed to *S. mitis* were compared by multiple *t* tests with correction. The dose‐dependent effect of nickel or titanium was compared using one‐way analysis of variance (ANOVA) followed by Bonferroni's multiple comparisons test. Differences were considered significant when *P* < .05. *P < .05; **P < .01; ***P < .001

Because TLR1, 2, 4, 5, and 6 are well‐known host receptors recognizing bacterial ligands, TLR 3 is also reported to be involved in skin sensitization^53^ as well as metal‐induced skin and mucosa irritation reactivity,^54^, ^55^ and all these TLRs may play a role in allergic reactions, for example, in asthma,^56^ we next determined the mRNA expression of TLR1‐6 in order to gain more mechanistic understanding of the differences observed between RHS and RHG upon *S. mitis* and metal exposure. The mRNA expression of TLR2, 4, and 6 was undetectable in all conditions (data not shown). In RHS, TLR1, 3, and 5 mRNA was not regulated (neither increased nor decreased) under any of the experimental conditions investigated (Figure 5).

Exposure of RHG to *S. mitis* (in contrast to RHS) resulted in increased TLR1 mRNA expression (Figure 5A). When *S. mitis* was co‐applied with nickel to RHG, a further increase in TLR1 mRNA expression was observed at the low concentration of nickel (3 mg/mL) followed by a decrease at the higher concentration (15 mg/mL) (Figure 5B). In contrast to TLR1, TLR5 mRNA levels were greatly decreased when RHG were exposed to *S. mitis* or the low concentration of nickel alone. When co‐applied, *S. mitis* and nickel resulted in a dose‐dependent increase in TLR5 mRNA. In contrast to nickel, titanium resulted in a trend for increased TLR3 mRNA levels both in the presence and absence of *S. mitis*. Lack of significance was most probably due to donor variation in the primary RHG cultures and low number of replicates (n = 3) in the complex organotypic model. TLR2, 4, and 6 mRNA levels were not regulated in RHG under any of the experimental conditions studied (data not shown).

**FIGURE 6:**
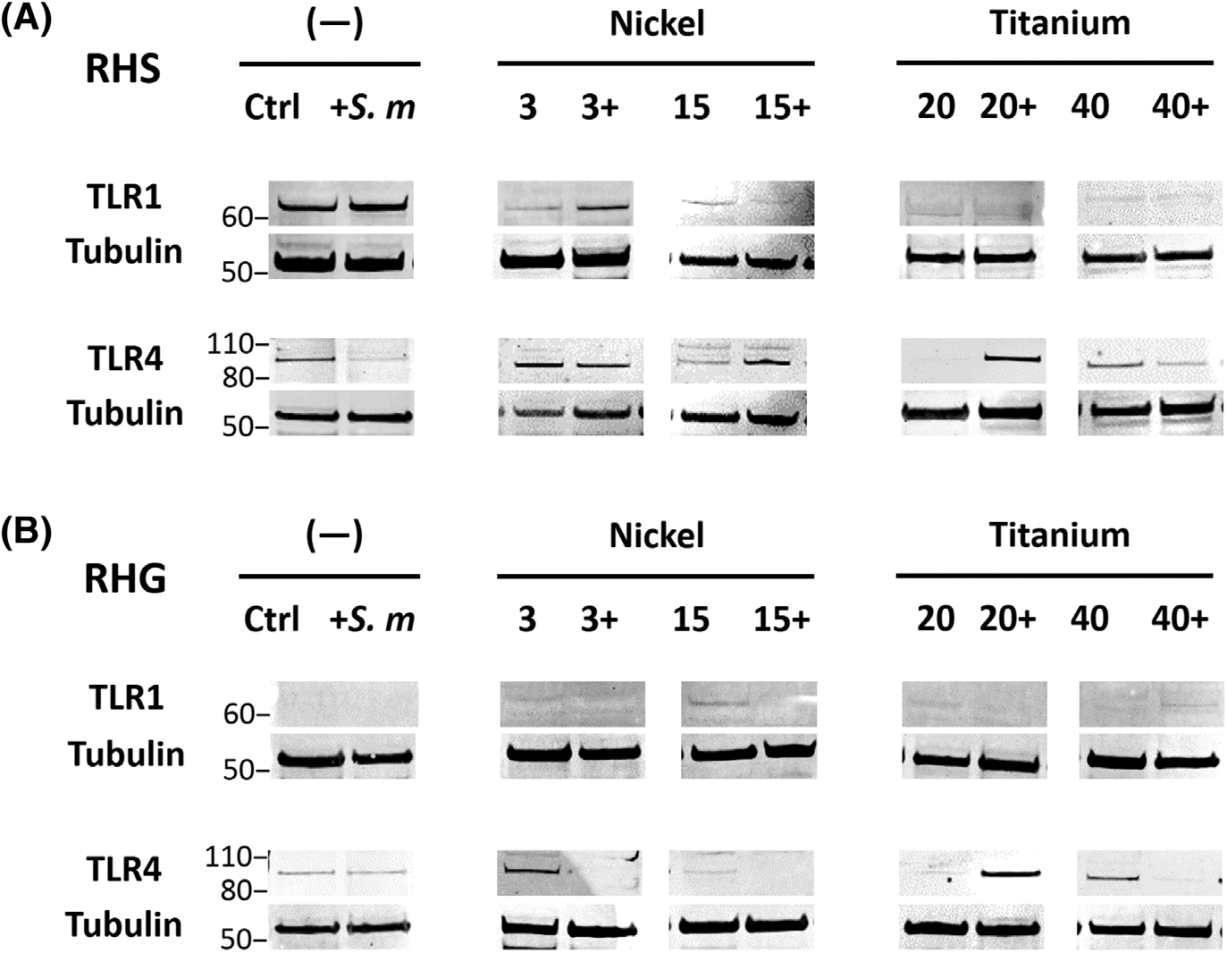
Toll‐like receptor (TLR) protein expression in reconstructed human skin (RHS) and gingiva (RHG). TLR1 and 4 proteins are shown together with reference tubulin expression. TLR2, 3, 5, and 6 were under the detectable level (data now shown). Data are representative of three independent experiments

Because mRNA levels give only an indication of gene activity and mRNA stability for a particular protein at a particular time point, we next determined TLR protein levels by western blot. Only TLR1 and TLR4 proteins were detectable and then only under certain experimental conditions (Figure 6); TLR2, 3, 5, and 6 proteins were undetectable in both RHS and RHG (data not shown). TLR1 protein was strongly detectable in unexposed RHS (Figure 6A). *S. mitis* alone did not influence this TLR1 expression, whereas nickel greatly reduced TLR1 expression. When co‐exposed with *S. mitis*, high nickel concentrations also reduced TLR1 expression. Titanium, either alone or co‐exposure with *S. mitis* resulted in pronounced clear decrease in TLR1 expression. In contrast, TLR4 expression was suppressed in RHS after *S. mitis* exposure, as well as metal exposure (concentration dependent). However, co‐exposure of *S. mitis* with nickel or titanium resulted in TLR4 protein being detectable again. Taken together, we show that for RHS, TLR1 and TLR4 protein was decreased or unaltered by *S. mitis* and/or metal exposure compared to unexposed RHS (Figure 6A).

In contrast to RHS, TLR1 protein levels were negligible in RHG with only a slight band being visible when RHG were exposed to nickel (Figure 6B). However, TLR4 protein was clearly expressed in RHG. *S. mitis* exposure did not influence TLR4 expression but both nickel and titanium exposure resulted in a moderate increase in protein. Notably, co‐exposure of *S. mitis* and nickel suppressed TLR4 protein levels, whereas co‐exposure with titanium (low concentration) increased TLR4 expression.

Taken together, these results indicate that *S. mitis* and metal exposure are able to differentially regulate TLR1 and 4 expression in RHS and RHG, with TLR1 and 4 being involved in RHS, and predominantly TLR4 being involved in RHG.

## 4 DISCUSSION

**FIGURE 7:**
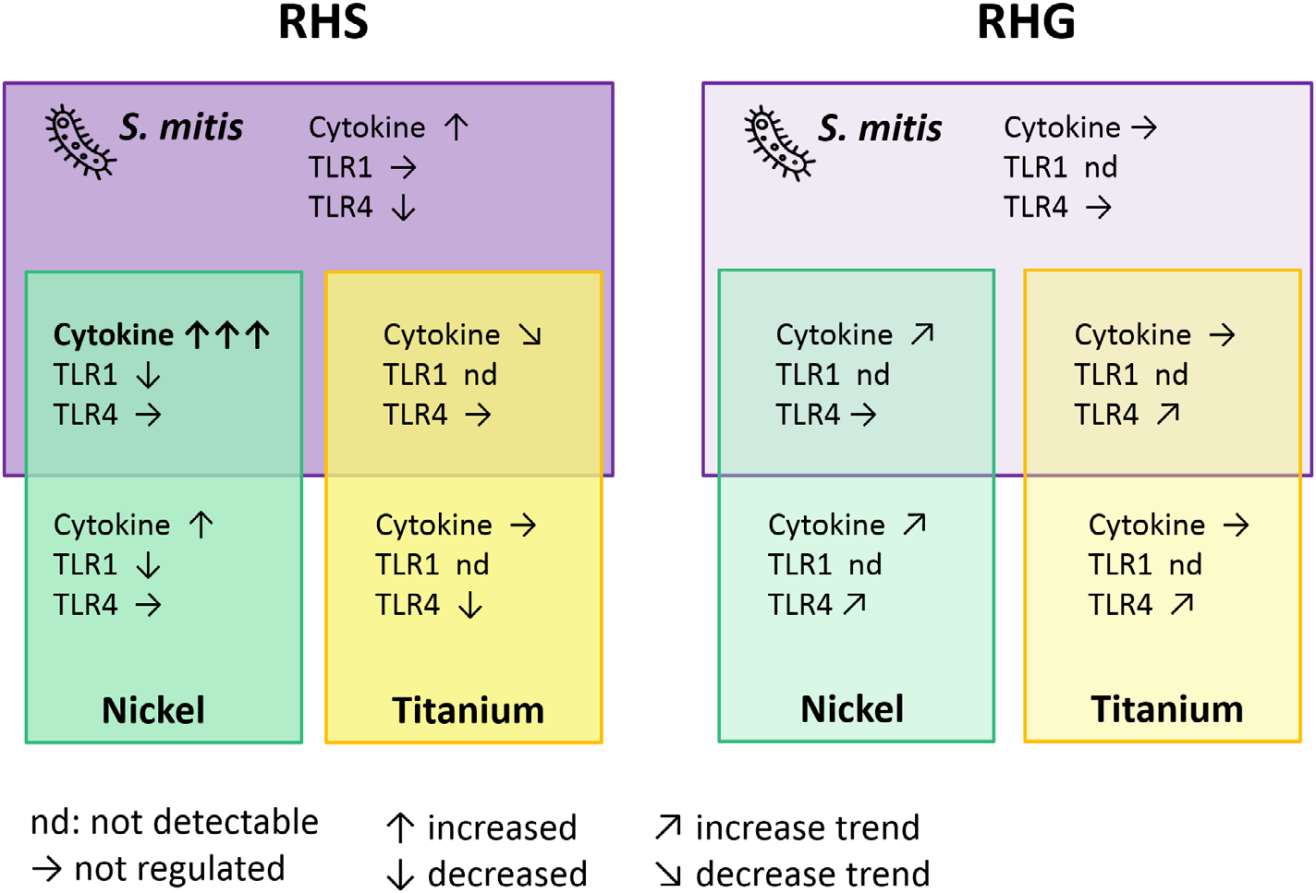
Schematic overview of the differential influence of *Streptococcus mitis* and metals on the host response in reconstructed human skin (RHS) and gingiva (RHG). The response of RHS and RHG exposed to *S. mitis*, nickel, and titanium is illustrated. The schematic overview summarizes data obtained from cytokine secretion (ELISA) and protein expression of Toll‐like receptors 1 and 4 (TLR1 and TLR4, western blot). The arrows show relative regulations compared to the corresponding vehicle‐exposed (Hank's Balanced Salt Solution, HBSS) culture. The overlapping areas in the middle represent the host response to co‐exposure of *S. mitis* and nickel or titanium

The aim of this study was to determine how commensal bacteria, *S. mitis*, influence the host response to metals in reconstructed human skin (or RHS) and gingiva (or RHG). Nickel and titanium were chosen for our study because nickel is a well‐characterized contact sensitizer,^2^, ^15^ whereas the sensitizing potential of titanium is still questionable.^32^ The RHS and RHG models were compared throughout our study, since clinical experience indicates that skin exposure leads to sensitization, whereas oral mucosa exposure leads to tolerance,^3^, ^4^ with the reasons for this still being unknown. Indeed, our results showed that co‐exposure of *S. mitis* and nickel resulted in a more potent innate immune response in RHS than in RHG. In comparison, titanium remained inert (see schematic overview, Figure 7). These results indicate the important roles of commensal microbes and route of exposure in the host response to metals.

*S. mitis* is a commensal bacteria common to both skin and gingiva.^46^, ^47^ Therefore it was important in our study to show no detrimental effects to either bacteria or host during our experiments. We ensured that exposure conditions were noncytotoxic for the RHS and RHG, whereas at the same time taking care that *S. mitis* remained viable for the duration of the study. Only very few studies have previously exposed RHG (but not RHS) to commensal microbes, and none have shown such well‐preserved tissue integrity after 24 hours of co‐culture. For example, a recent study described gene regulation in RHG after *Streptococcus oralis* biofilm exposure via a titanium implant inserted into the RHG for 24 hours; however, no data on tissue viability and limited data on tissue integrity were shown.^57^ Another study described biofilm formation and invasion into the epithelial layers when exposed to streptococci and *Candida albicans* for 24 hours. However, this study also showed an increased number of caspase‐3 positive cells, strongly suggesting a significant decrease in tissue viability.^58^

Previously we have described intrinsic differences between skin and gingiva with regard to innate immunology, wound healing, and the response to contact sensitizers.^48^, ^59^, ^60^, ^61^ In current study, under unexposed conditions, we show that RHS secretes higher baseline levels of cytokines involved in LC migration (IL‐18 and CXCL12) than RHG, which is in line with our previous study,^25^, ^48^ and we also show that *S. mitis* increased the cytokine release of IL‐6, CXCL8, CCL5, and CCL20 in RHS but not in RHG. Such relative inertness of RHG to *S. mitis* is supported by findings of others describing the regulatory role of *S. mitis* as an oral commensal on immune balance, where it inhibits CD^4+^ T cell proliferation, promotes Th17 responses, and induces the secretion of IL‐10 in human monocytes.^12^, ^62^ To our knowledge, no reports describe how *S. mitis* affects the skin. Taken together, our results and those of others support the characteristic of oral mucosa in balancing a potential overactivated immune response induced by commensals. This is necessary for maintaining oral health because the oral cavity is continuously exposed to food antigens, sensitizers, and abundant microorganisms, for example, viruses and fungi^11^, ^63^ in addition to commensal bacteria. The undetectable levels of IL‐10 in our study could be due to the absence of immune cells in the RHS and RHG, as it has been reported that commensal bacteria do induce IL‐10 in murine dendritic cells^64^ and that human monocytes and lymphocytes produce IL‐10 .

This article describes in detail the results obtained from foreskin keratinocytes. Our previous study using adult abdominal skin showed secreted protein concentrations falling within the ranges observed for the foreskin donors in all cases.^48^ This indicates that as far as the secreted proteins described in this article are concerned, there are no differences between donor age or between child foreskin and adult abdominal skin. This is further supported by our previous finding that RHS can be constructed reproducibly from donors 4 to 90 years of age in a 3‐week culture period and show no variation in proliferation or migration.^65^ This is particularly surprising, since in the literature, studies describe clear differences between neonate and adult skin but with regard to melanocytes,^66^ epidermal LC,^67^, ^68^ innate immune cells, for example, neutrophils and monocytes^69^ and T cells,^70^ which are all absent in our model, which contains only keratinocytes and fibroblasts.

Notably, our results showed that the commensal *S. mitis* strongly enhanced the innate immune response to nickel in RHS, especially the release of CXCL8. In contrast, the *S. mitis–* and nickel‐mediated cytokine response in RHG was considerably lower than in RHS, in line with a more tolerogenic environment (Figure 7). These current findings are in line with our previous findings and those of others who showed that CXCL8 was induced when DCs, macrophages, and keratinocytes were exposed to nickel.^34^, ^54^, ^71^ *S. mitis* was found previously to induce CXCL8 in oral epithelial cells^72^: however, no further studies have shown its influence on RHG or on skin. Nickel ions are sensitizers that are known to be able to penetrate the body barriers and activate innate immunity. At a cellular level, this leads to the upregulation of signaling pathways: the Nuclear factor kappa‐light‐chain‐enhancer of activated B cells (NF‐κB) pathway, the Mitogen‐Activated Protein Kinase (MAPK) pathway and the NLRP3 inflammasome in, for example, keratinocytes and LCs, which ultimately results in the release of cytokines and chemokines of various functions.^2^, ^15^, ^73^ This innate immune response is crucial for triggering the T cell–mediated adaptive immune response, which is a key event in the sensitization process, especially where weak or moderate sensitizers are concerned, since the innate response contributes to the threshold of activation level in the host.^74^ Upregulation of the inflammasome and the resulting cytokine/chemokine cascade can promote sensitization by (a) recruiting immune cells (eg, CXCL8, CCL2 secreted by keratinocytes^71^, ^75^); (b) inducing leukocyte adhesion to endothelial cells (Vascular Cell Adhesion Molecule 1 (VCAM1), Intercellular Adhesion Molecule 1 (ICAM1), E‐selection secreted by keratinocytes and endothelial cells^15^); and (c) activating other signaling molecules that regulate the immune response during contact hypersensitivity (eg, IL‐12 in mice^76^). The threshold for sensitization is currently thought to be not only tightly regulated by the activation and maturation state of DCs but also by the amount of cytokines secreted by local DCs, keratinocytes, and fibroblasts,^6^, ^7^ which are influenced by the local commensal microbes at the same time. Our observation that co‐exposure of *S. mitis* and nickel further increases cytokine release would suggest that such a co‐exposure would increase an individual's chance of sensitization. Furthermore, introducing co‐exposure methodology, would be expected to increase the sensitivity of in vitro assays.

In contrast to nickel, titanium suppressed *S. mitis*–induced cytokine secretion in RHS and had no influence on RHG, strongly indicating that titanium is inert (Figure 7). Indeed titanium implants have been reported to cause allergic manifestations in clinic,^26^ but these findings are still questionable due to the limited in vivo studies and inconclusive results from in vitro studies.^21^, ^26^, ^32^ In general, titanium is suggested to be harmless and a nonsensitizer on skin: TiO_2_ cannot penetrate the skin stratum corneum^32^; TiO_2_ nanoparticles (TiO_2_NPs) may^77^ or may not^32^ penetrate skin epidermis. TiO_2_NP is also inert for THP‐1 macrophages because it does not influence genes involved in modulating macrophage maturation, inflammatory responses, chemotaxis, and leukocyte migration.^78^ In addition, TiO_2_ cannot induce skin sensitization in LLNA in mice.^79^ However TiO_2_NPs were found to act as an adjuvant when co‐applied with the bacterial fragments LPS and peptidoglycan;^80^ or co‐applied with ovalbumin (OVA)^28^ or toluene‐2,4‐diiscocyanate (TDI),^81^ which significantly induced pulmonary sensitization in mice. This is in line with the fact that co‐application of an irritant with a sensitizer will increase the sensitization potential of the sensitizer.^82^ Our findings in this study, together with our previous studies with reconstructed human epidermis,^21^ further support titanium as being an inert, nonsensitizing metal that may have weak irritant properties under certain conditions.

Our results showed that *S. mitis* and metals differentially regulate TLR1 and 4 in RHS, and predominantly TLR4 in RHG (Figure 7). Certain exposure conditions resulted in TLR4 no longer being visible. This absence of TLR4 is most probably due to it becoming internalized into the endosome, which allows further activation of the intracellular TRIF‐related adaptor molecule (TRAM)‐TIR‐domain‐containing adapter‐inducing interferon‐β (TRIF) pathways^83^ and finally results in generating the innate immune signal (cytokine release; eg, CXCL8) that leads to functional innate immune responses.^38^, ^84^ TLR4 is a versatile and complex host receptor that is involved in both host‐sensitizer and host–microbe interactions, keeping the skin alert and oral mucosa tolerant. Whereas it helps to enforce the tolerogenic properties of oral LCs against the LPS‐derivative MPL (monophosphoryl lipid A),^15^, ^85^ it notably plays a crucial role in nickel allergy.^23^, ^86^ Nickel ions can bind directly to the conserved histidines of TLR4, resulting in TLR4 dimerization and the initiation of the cytokine‐release cascade.^87^ The minimum amount of nickel to induce skin sensitization in transgenic mice expressing human TLR4 was reduced when nickel was co‐applied with LPS, and both the sensitization and elicitation steps during nickel allergy coincided with activation of TLR2 and TLR4, suggesting a potential role of bacteria exposure as well as nickel in inducing skin sensitization.^15^, ^35^, ^40^, ^45^, ^88^ However, in vitro studies showed that only TLR4 and not TLR2 was activated when human keratinocyte cultures were co‐exposed to nickel and LPS,^34^ whereas another suggested that TLR2 could participate in nickel activating innate immunity in lung fibroblasts.^33^ This discrepancy may be explained by species‐specific differences in TLR4 between human and mouse,^23^ and might also be dependent on the site of exposure, as no such co‐application has been studied on human skin or on human oral mucosa. Previously we have exposed RHG to multi‐species–commensal oral biofilm derived from saliva, and we have found in line with this study the presence of TLR4 protein and absence of TLR1 and TLR3 proteins.^38^ However, in contrast to this study, the commensal biofilm resulted in a large increase in cytokine secretion from RHG, and TLR2 was strongly expressed.^89^ The differences in these two studies can most likely be attributed to the presence of bacteria other than *S. mitis* in the multi‐species oral biofilm.

Of interest, although TLR1 protein was not detectable in RHG exposed to any conditions, *S. mitis* alone or in combination with nickel resulted in an increase in mRNA levels, indicating that exposure resulted immediately in increased transcription and may increase protein levels at time intervals longer than the 24‐hour exposure period of our study. Notably, upon exposing to titanium, the expression of TLR1 mRNA was not upregulated and the TLR1 protein was undetectable in both RHS and RHG, which is in line with a previous study showing that titanium particles exposure was associated with low TLR1 protein expression in mice bone and periosteal cells.^36^ Similarly, TLR5 mRNA was present in RHG, although the protein was not (yet) detectable. Taken together, our results for TLR1 and TLR4 visualize the balance between surface‐expressed TLR, ligand‐bound internalized TLR, and transcription/translation of TLR mRNA.

Limitations of our study should also be considered. Immune cells (eg, LCs) have important roles in sensitization and in host–microbe interactions; however, they are absent in the present study. Furthermore, we used only a single bacteria species for the exposure, whereas in vivo, a broad variety of other microbial species (including potential pathogens) are also in contact with the skin and the oral mucosa. The influence from these on host tissues, especially the pathogens, may provide more information to interpret conflicting results from in vitro and in vivo studies. In addition, sensitization occurs on host tissues, which are already influenced and primed by local commensal microbes, whereas in this study *S. mitis* and metals were co‐exposed to sterile RHS and RHG. However, this first study does show that microbes may promote the innate immune response to nickel in skin considerably more than in oral mucosa, thus shedding light on the mechanism between skin sensitization and oral tolerance to nickel. Furthermore, our study continues to support titanium as being a nonsensitizing metal.

## AUTHOR CONTRIBUTIONS

**Lin Shang:** Conceptualization; data curation; formal analysis; funding acquisition; investigation; methodology; writing‐original draft; writing‐review and editing. **Dongmei Deng:** Conceptualization; data curation; formal analysis; methodology; supervision; writing‐review and editing. **Sanne Roffel:** Data curation; formal analysis; investigation; methodology; writing‐review and editing. **Susan Gibbs:** Conceptualization (equal), funding acquisition (equal), project administration (lead), resources (lead), supervision (lead), validation (equal), writing‐review & editing (equal).

## CONFLICT OF INTEREST

Susan Gibbs is scientific advisor for Geneus BioTech CRO (SME). The other authors have no interests to declare.
